# Genetically modified mesenchymal stromal cells in cancer therapy

**DOI:** 10.1016/j.jcyt.2016.09.003

**Published:** 2016-11

**Authors:** Elizabeth K. Sage, Ricky M. Thakrar, Sam M. Janes

**Affiliations:** 1 Lungs for Living Research Centre, UCL Respiratory, Rayne Institute, University College London, London, United Kingdom; 2 Department of Thoracic Medicine, University College London Hospital, London, United Kingdom

## Abstract

The cell therapy industry has grown rapidly over the past 3 decades, and multiple clinical trials have been performed to date covering a wide range of diseases. The most frequently used cell is mesenchymal stromal cells (MSCs), which have been used largely for their anti-inflammatory actions and in situations of tissue repair and although they have demonstrated a good safety profile, their therapeutic efficacy has been limited. In addition to these characteristics MSCs are being used for their homing and engraftment properties and have been genetically modified to enable targeted delivery of a variety of therapeutic agents in both malignant and nonmalignant conditions. This review discusses the science and technology behind genetically modified MSC therapy in malignant disease and how potential problems have been overcome to enable their use in two novel clinical trials in metastatic gastrointestinal and lung cancer.

## Introduction

The landscape of cellular therapies has changed dramatically over the past 20 years and is likely to continue to do so over the next decade. There is an increasing drive to overcome existing roadblocks to large-scale use to provide a more streamlined route to market. The value of the cell therapy industry is projected to reach £20 billion by 2022, and the array of cell therapies being investigated is rapidly expanding [1]. There are currently more than 500 clinical trials using mesenchymal stromal cells (MSCs) registered on the National Institutes of Health clinical trials database and an increasing proportion of these are using genetically modified MSCs (http://www.clinicaltrials.gov; accessed August 2016). In the United Kingdom alone 37% of trials use genetically modified cells, the majority of which use viral vectors for gene delivery [2].

The term “cell therapy” covers a wide array of products, and they are most commonly classified according to cell type (e.g., hematopoietic stem cells, MSCs, embryonic stem cells, modified T cells). Within these cell types, the range of diseases being treated are vast, ranging from immunomodulation to target inflammatory diseases such as inflammatory bowel disease [3], chronic obstructive pulmonary disease [4] and acute lung injury [5], [6] to acute stroke [7], [8], acute myocardial infarction [9], [10], and graft-versus-host disease [11]. Stem cells are also being used for tissue repair and regeneration with differentiation being directed to the target organs such as bone and cartilage [12]. There is also increasing interest in the use of genetically modified cell therapies including chimeric antigen receptor (CAR) T cells and cells genetically modified to express therapeutic proteins targeted to a specific disease.

Within this review we discuss the use of genetically modified MSCs as a therapy for cancer and in particular discuss our own experience of developing and cell and gene therapy product for the treatment of metastatic lung cancer to be delivered in a phase I/IIa clinical trial.

## MSCs

MSCs were first described in the 1970s by Friedenstein *et al.* [13] and are now one of the most widely characterised adult stem cells. As determined by the International Society for Cellular Therapy (ISCT), they must meet the minimum criteria of being adherent to tissue culture plastic under standard culture conditions, express the cell surface markers CD105, CD73 and CD90 and lack expression of CD45, CD34, CD14 or CD11b, CD79α or CD19 and HLA-DR surface molecules. In addition, they must be capable of differentiating into adipocytes, osteoblasts and chondroblasts under the correct experimental conditions [14]. MSCs are a heterogeneous population of cells, and their characteristics are affected by passage, cell density and culture conditions [15]. They are readily available from multiple sources, including bone marrow [16], adipose tissue [17] and umbilical cord [18], among others, and although cells from all sources will meet the minimum criteria for MSC definition, there are subtle differences in their behavior that further complicates our understanding of this cell type. These differences may be therapeutically beneficial in some cases in terms of either their secretory profile or growth characteristics, but as yet there are no data that directly compare the core characteristics of the different sources of MSCs, and the ideal cell source is likely to be dependent on the indication for its use.

MSCs can be easily extracted from adults and expanded *in vitro* and, once isolated, have a number of characteristics that make them appealing vectors for delivery of therapeutic agents. One of the key properties of MSCs is their tumor tropism, that is, their propensity to move toward sites of tumor [19], [20]. The precise mechanism through which this process occurs is unknown, but it has been demonstrated in multiple cancer models including glioma [21], [22], breast carcinoma [23], lung cancer [24], [25], malignant mesothelioma [26], hepatocellular carcinoma [27], [28], colon cancer [29], pancreatic cancer [30], [31], ovarian cancer [32], melanoma [33] and Kaposi sarcoma [34]. The tropism is thought to be mediated through paracrine signaling between the tumor microenvironment and corresponding receptor expression in MSCs. Although tumor tropism has been consistently demonstrated, the precise mechanisms responsible remain poorly understood. Many factors have been assessed with regards to this property including multiple receptors, extracellular matrix proteins, tumor necrosis factor α (TNFα), interleukins (ILs), macrophage migration inhibitory factor (MIF) and, most frequently, the soluble tumor–derived factor stromal-derived factor (SDF)-1 [35], [36], [37]. The most widely studied interaction has been that between SDF-1 and CXCR4, but the involvement of this axis remains controversial [38].

Another characteristic of MSCs that make them attractive for therapeutic use is their low immunogenic state in that they elicit a weak allogeneic immune response when delivered to a non-identical, non-matched recipient [39], [40]. These unique properties are attributed to the low levels of expression of major histocompatibility complex (MHC) class I and the co-stimulatory molecules CD80 and CD86 and the lack of MHC class II proteins [41], [42], [43], [44]. Because of these properties, there is the potential for using allogeneic MSCs as an “off-the-shelf” product. To use cells from healthy, young donors that are likely to have greater regenerative capacities and higher proliferative rates would be an attractive option to help control the costs and complexity of the manufacturing process, which would be a significant factor in the long-term likelihood of making cell therapies commercially viable. Although there is evidence that the source of MSCs and their culture conditions can alter their immunomodulatory properties, there is no direct comparison of the immune profile of cells from different sources or after culture in different conditions [45], [46].

## Genetic modification

Going hand in hand with their tumor tropism is the ability of MSCs to be modified to allow sustained delivery of specific anti-cancer agents. Because the cells are attracted to tumor stroma, targeted therapeutic delivery can be achieved at multiple tumor sites. There are many methods to genetically modify MSCs, but they can be broadly classified into viral and non-viral methods. A detailed discussion of methods of modification and MSC engineering is outside the scope of this review; however, excellent overviews of this are provided by Park *et al.* [47] and others [48], [49].

### Non-viral vectors

Non-viral methods of gene transfer encompass all physical and chemical methods of gene delivery. These methods are appealing because they are able to deliver larger transgenes than viral methods, are more cost-effective and are amenable to scale-up manufacturing and induce less of an immune response. Despite these benefits, there are a number of limitations, the main one being their low transfection efficiencies and transient gene expression [47]. Physical methods of gene delivery are based on temporarily penetrating the cell membrane using techniques such as electroporation [50], [51], [52], [53], ultrasound [54], [55], magentofection [56] and DNA particle bombardment by gene gun [57], [58]. Chemical methods tend to use cationic lipids or polymers, which form negatively charged particles that are taken up into the cell by endocytosis, but these methods are largely limited to *in vitro* use [59], [60]. Cell surface receptors have been explored, and other non-viral methods of modification being investigated are via liposomes [61] or nanoparticles [62].

### Viral vectors

Viral transduction of MSCs is commonly achieved using lenti-, retro-, adeno- or adeno-associated virus without affecting their stem cell properties [63], [64]. Viral vectors use the innate ability of the virus to gain entry into and survive within the host cell nucleus to ensure continued expression of the viral genome. To make them useful as delivery vectors, they have undergone significant modification to produce replication incompetent viruses with attenuated cytopathic effects and immunogenicity. One of the enduring concerns regarding the use of viral vectors is their safety, but advances in vector design have helped to alleviate this matter [65], [66], [67], [68], [69].

Each type of viral vector has its pros and cons (Table I), and the choice of vector used will be dependent on the therapy required and the disease being treated. Viral vectors are particularly appealing because they enable high transduction efficiency and, depending on the type of virus used, can deliver long-term stable transgene expression. The choice of genetic modification will be determined by the aim of the therapy. Some genetic modification is designed to improve homing by overexpression of key chemokines such as CXCR4 [70] and epidermal growth factor receptor [71], and others aim to deliver a specific therapeutic protein, such as the pro-apoptotic molecule TNF-related apoptosis inducing ligand (TRAIL) [25], [26], [72]. Other modifications being assessed for therapeutic efficacy in a variety of pre-clinical disease models include interferon (IFN)-β (IFNβ) [21], [33], [73], [74], IL-12 [75], [76], IFN-ɣ [77], angiopoietin 1 [78], endothelial nitric oxide synthase [79] and vascular endothelial growth factor (VEGF) [80].

## Genetically engineered MSCs as cancer therapeutics

Cancer is a devastating disease, and the number of people diagnosed every year is on the increase. Fifty percent of people born after 1960 are likely to be diagnosed with some form of cancer during their lifetime, and breast, prostate, lung, and bowel cancers were responsible for more than 50% of the cancer diagnoses in the United Kingdom in 2013. For all cancers, the 10-year survival rate is 50%, but this is highly variable depending on the specific cancer subtype. Lung cancer has a particularly poor prognosis with an incidence of >45 000 in the United Kingdom alone in 2013 and more than 35 000 deaths. The 10-year survival is only 10%, a figure that has not changed significantly over the past 40 years. Breast cancer, on the other hand, fares much better with >53 000 new diagnoses a year with a 78% 10-year survival; that of prostate cancer is even better with an 84% 10-year survival rate [81].

Regardless of the type of cancer, there are three main categories of treatment: surgery, chemotherapy and radiotherapy. Surgery is often the only curative treatment, but many patients will have to undergo either chemotherapy, radiotherapy or both in addition to surgery. For those in whom surgery is not an option, the mainstay of treatment is usually chemotherapy, particularly for those with advanced disseminated disease. The main problem with existing chemotherapy agents is their toxicity, with the most common side effects being marked gastrointestinal upset, such as nausea and vomiting, and bone marrow suppression making patients susceptible to overwhelming infection and in some cases death.

With the significant side effect profile of traditional chemotherapeutics, novel cancer treatments are needed and a number of groups are looking to capitalize on the tumor tropic properties of MSCs to develop targeted anti-cancer therapies using MSCs as delivery vehicles.

### Pre-clinical therapies

A number of pre-clinical studies have looked at the efficacy of genetically engineered MSCs in a wide range of malignant diseases. These have used MSCs from a variety of sources, different transfection methods for gene delivery, multiple different transfected products and a wide range of tumor models, but despite these variations, the data have consistently shown a reduction in tumor growth and prolonged survival (Table II).

One of the first studies exploring the use of genetically modified MSCs in cancer transduced human MSCs with INF-β and injected them to treat a murine xenograft model of melanoma, resulting in a reduction in tumor growth and increased survival in treated animals [33]. This approach has subsequently been used in models of breast cancer and glioma with similar beneficial effects [21], [73]. Following from this, other groups have looked at developing therapeutics aimed at targeting cell proliferation using IL-12 [82], angiogenesis with VEGFR-1 [83] and pigment epithelium-derived factor (PEDF) [84] and nitric oxide synthase [85].

Other therapeutics are aimed at inducing cancer cell apoptosis. TNF-related apoptosis inducing ligand (TRAIL) is a transmembrane protein that acts via death receptors to activate the extrinsic apoptotic pathway resulting in apoptosis of cancer cells without affecting healthy cells. Current chemotherapy agents act via the intrinsic pathway that senses DNA damage and again triggers downstream apoptosis. Because there is cross-talk between the two pathways, combining TRAIL therapy with chemotherapy results in a synergistic treatment suggesting that TRAIL could be used in conjunction with current first-line clinical therapies [86], [87], [88]. Pre-clinical work using MSCs modified to express different forms of TRAIL have been shown to have therapeutic efficacy in pre-clinical models of mesothelioma [26], [72], lung metastases [25], breast cancer [89], cervical cancer [90], myeloma [91] and glioma [22]. Another apoptotic protein, apoptin, has similar properties and has been used against hepatocellular carcinoma [92].

Other novel approaches to cancer treatment are the delivery of suicide genes via MSCs. The premise behind this therapy is that MSCs engineered to express suicide genes are delivered into tumors and are activated once treatment with systemic chemotherapy agents is given. This approach has been demonstrated largely in glioma using MSCs expressing cytosine deaminase/uracil phosphoribosyltransferase (CDy/UPRT), which is activated after treatment with 5-fluorocytosine (5-FC) [93] and MSCs expressing thymidine kinase, which is subsequently activated with ganciclovir [94].

Many of these agents are attractive for delivery via MSCs because their use as anti-cancer agents after systemic delivery is limited by short half-lives or excessive systemic toxicity [95]. For some agents, the concentrations required to result in a therapeutic effect would be significantly higher than levels achieved following intravenous systemic administration at a tolerated dose [96], [97], [98]. Enabling delivery of the agent directly into the tumor would allow long-term low-dose protein expression without the toxicities seen with systemic delivery.

### Therapies in clinical trials

With so many pre-clinical studies showing the *in vitro* efficacy of many types of genetically modified cell therapy, it is perhaps not surprising that the next step is assessing the safety and efficacy of these therapies in the clinical trial setting. Looking at the literature, there are more than 500 clinical trials looking at the safety and efficacy of MSCs from either allogeneic or autologous sources, and there is overwhelming evidence of safety. From an efficacy perspective however the results have been largely disappointing. The majority of clinical trials have been in the treatment of inflammatory conditions such as chronic obstructive pulmonary disease [4] and adult respiratory distress syndrome [5], [99], but regardless of the disease being treated, they all use unmodified MSCs for their immunomodulatory and anti-inflammatory properties.

To date there have been no clinical trials looking at the delivery of genetically modified MSCs in patients with cancer; however, this landscape is set to change with two first-in-human clinical trials assessing genetically modified MSCs in gastrointestinal cancer and lung cancer. There are a number of challenges when translating this type of cell therapy into the clinic, in particular around manufacturing a clinical-grade product. Cell and gene therapies differ from traditional biopharmaceuticals in that they are inherently a heterogenous living product, the characteristics of which can be affected by multiple variables, such as the culture media, conditions of hypoxia versus normoxia, adherent versus spheroid culture and any changes in process that need to be performed to achieve a large-scale expansion while remaining cost-effective. To ensure products retain their efficacy, any changes in conditions require evidence that both the MSC function and that of the therapeutic protein remain unaffected. Another of the great unknowns regarding cell therapies is the fate of the cells after intravenous delivery. It is possible that the clinical trials to date have shown limited efficacy because the cells are quickly removed from the body. To highlight the challenges and rationale behind clinical trial design for these products, the following sections discuss in more detail the two ongoing trials using genetically modified cell therapies for cancer.

## TREAT-ME1 trial for gastrointestinal tumors

Adenocarcinomas of the gastrointestinal system account for significant morbidity and mortality, and, as with many cancers, treatment is complicated by high rates of tumor recurrence, resistance to chemotherapies and the presence of locally advanced disease that is not amenable to surgical resection [100]. These malignancies share common morphologic characteristics regardless of the tissue of origin, in particular the presence of a tumor stromal microenvironment that is permissive to metastasis formation [101]. Attempts to target novel cancer treatments to components of the microenvironment in a bid to suppress tumor growth and metastases have shown pre-clinical efficacy [102], and in particular groups have looked to harness the tumor tropic ability of MSCs to develop new anti-cancer therapeutics.

### Pre-clinical rationale

Once MSCs are recruited to the tumor microenvironment, they induce the expression of the chemokine CCL5/RANTES, which causes increased tumor neo-vascularization and aids the recruitment of other stromal cell types to encourage tumor growth. Zischek *et al.* [31] used this mechanism to design a genetically modified cell therapy that would be attracted to the tumor stroma and activated by the presence of CCL5 to release a suicide gene resulting in tumor cell death. To achieve this, they stably transfected MSCs with a retroviral vector expressing thymidine kinase of the herpes simplex virus (*HSV-Tk*) under the control of a CCL5/RANTES promoter and delivered it intravenously to an orthotopic pancreatic tumor model. After cell delivery, animals received the pro-drug ganciclovir, which is phosphorylated by the HSC-Tk and drives cells into apoptosis. Delivery of these genetically modified cells resulted in a reduction in tumor growth and metastasis formation [31]. The same cells were tested in an orthotopic model of hepatocellular carcinoma with a similar therapeutic outcome [103].

By exploiting the biological activity of the tumor microenvironment, it has been possible to develop a genetically modified cell therapy that is only activated within the tumor deposit in response to the presence of a selectively expressed chemokine. Activated cells can then be used to initiate a pro-apoptotic process resulting in the selective death of cancer cells. By combining these properties, this novel cancer therapy should have high efficacy with few side effects.

### Clinical trial design

Continuing on from their pre-clinical work, TREAT-ME 1 is a prospective, uncontrolled, single-arm phase I/II study to assess the safety and efficacy of autologous MSCs genetically modified with a retroviral vector expressing tyrosine kinase and subsequent ganciclovir infusions in patients with gastrointestinal adenocarcinoma [104] that is currently recruiting patients.

In this first-in-human study, the investigating group has joined with a commercial partner, Apceth, to develop the investigational medicinal product (IMP) MSC_apceth_101. This uses autologous BM-MSCs that are isolated and expanded to passage 1 and subsequently transduced using a gamma-retroviral SIN-vector to express HSV-Tk under the control of the RANTES promoter. To ensure a pure population after transduction, cells are selected using puromycin and then expanded to generate the clinically required dose before being cryopreserved. To release the IMP, certain criteria need to be met, and these should reflect the safety and efficacy of the product. For this product, >90% of cells should express MSC markers and cell viability should be >80% with >75% of cells positive for the transgene and evidence of adequate transgene expression as determined by the sensitivity to ganciclovir.

When considering standard clinical trial design, most phase I studies are dose-escalation studies, and this trial is no different. Patients will receive three IMP infusions dosed according to body weight 1 week apart followed by ganciclovir given on 3 consecutive days starting 48–72 h after IMP delivery. Two doses will be tested, 0.5 × 10^6^ and 1 × 10^6^ cells/kg per dose. Assuming no dose-limiting side effects, the study will proceed to phase II. Phase II will treat 16 patients with adenocarcinoma of the gastrointestinal tract who will be divided into two groups: relapse/progression of disease or patients eligible for neoadjuvant therapy before surgery in which the IMP will be delivery 48–72 h before surgery as a single dose and ganciclovir on days 1–3 post-operatively. The primary end point of the trial is safety and tolerability of the IMP with secondary end points of tumor size and total number of metastases by Response Evaluation Criteria In Solid Tumors (RECIST) criteria. Additional information regarding the localization of the MSCs after delivery will be collected using the group of patients who will receive the IMP therapy immediately pre-operatively because this will enable detection of the therapeutic transgene in both resected tumor samples but also normal tissue adjacent to the tumor. This will start to provide key information to help address some of the unknowns regarding cell and gene therapy, in particular location and evidence of therapeutic gene expression and the results will be eagerly awaited.

## TACTICAL trial for lung cancer

### Pre-clinical rationale

Lung cancer is the leading cause of cancer death worldwide, and about 80% of lung cancer patients have a non–small cell histological subtype. Although early disease can be surgically resected with a curative outcome, the majority of patients present with advanced incurable disease [105]. For this patient population, chemotherapy with cisplatin and pemetrexed offers a survival benefit over active symptom control, but this benefit is small with an increase in median survival of 1.5 months [106].

One of the key benefits of using MSCs is to harness their tumor tropic effect to enable targeted delivery of anti-cancer therapies. TRAIL is an attractive cancer therapeutic because it selectively induces apoptosis in cancer cells without affecting healthy cells, although the precise mechanism through which this occurs is not clearly defined. TRAIL works by activating the extrinsic apoptotic pathway by binding to cell surface death receptors, whereas existing chemotherapies trigger the intrinsic apoptotic pathway by causing DNA damage. Because there is significant crosstalk between these two pathways, there is the potential of a synergistic effect of TRAIL with existing chemotherapy agents [87], [107]. In pre-clinical work, we have shown that MSCs can be successfully transduced with a lentiviral vector expressing TRAIL with transduction efficiencies of great than 90%. These TRAIL transduced MSCs can home to tumors and induce apoptosis, resulting in a reduction in tumor growth in both a lung metastases and mesothelioma model [25], [26].

### Clinical trial design

To follow on from this work, we are in the process of setting up the TACTICAL (TArgeted stem Cells expressing TRAIL as a therapy for lung CAncer) trial. This is a multicenter, prospective, randomized phase I/II trial to assess the safety and efficacy of third-party allogeneic MSCs transduced to express TRAIL as a first-line therapy in conjunction with chemotherapy in patients with metastatic adenocarcinoma of the lung. Currently the product is undergoing manufacture according to Good Manufacturing Practice criteria, and this has highlighted some of the challenges faced when manufacturing this kind of therapy at large scale.

### Cell manufacture and release

**Figure 1:**
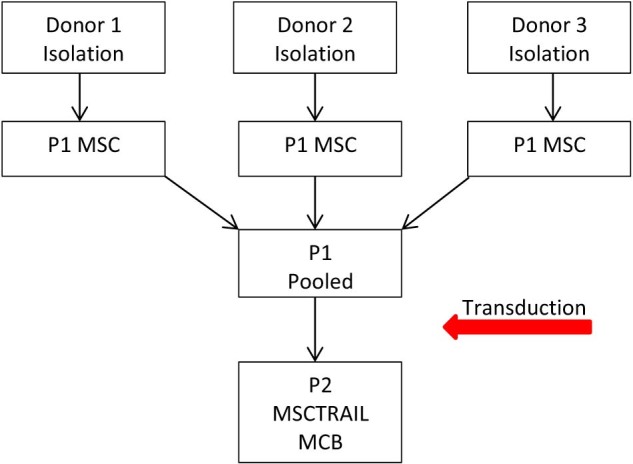
Schematic diagram showing the proposed manufacturing outline of the TACTICAL trial. MSCTRAIL, TRAIL-transduced MSCs.

Although MSCs are easy to isolate and expand *in vitro* reaching up to 50 population doublings, to ensure maximum efficacy and safety in the clinical setting, this figure is kept below 20 [108]. The majority of clinical trials using unmodified MSCs have used in the range of 1 × 10^6^ to 5 × 10^6^/kg and often use multiple doses, meaning the number of cells required per patient can reach as high as 10^8^. To produce the number of cells required and remain cost-effective, the best way to meet demand is to manufacture a master cell bank (MCB) of allogeneic cells that can be cryopreserved and expanded as required to make a working cell bank (WCB). This is the approach the TACTICAL trial is taking, and both to ensure maximum production capacity and to reduce the impact of the inherent variability of MSCs, we are pooling cells from multiple donors before transduction (Figure 1).

Both the MCB and WCB will be cryopreserved so that samples can be taken for stability assays and release criteria. Although most of the clinical trials using allogeneic MSCs have thawed the therapeutic product at the bedside, there has been some discussion in the literature that cryopreservation can affect certain key therapeutic characteristics of the cell product. We have already modified our cryopreservation procedure to ensure that our product is not affected, but this should be checked for any cell and gene therapy product that requires freezing [109].

To be fully compliant with the regulatory requirements set out by the European Medicine Agency, release criteria must be established and confirmed on both the MCB and WCB. These criteria are based not only on the basic characteristics required to confirm the MSC identification, viability, and sterility but also those required to ensure that the product demonstrates its potency and efficacy for its intended clinical application. This can be particularly challenging for untransduced MSCs whose mode of action is not always clearly defined and to attempt to address this and create a gold standard, the International Society for Cellular Therapy has recently published guidelines on standardized immune functional assays to demonstrate MSC potency [110]. This is an area where modified MSCs have an advantage in that the gene modification is usually what determines therapeutic potency and is often a much more objective readout.

### Cell localization

Another great uncertainty regarding MSC therapy is the fate of the cells after intravenous injection. Although there is a wealth of pre-clinical data using a wide variety of optical imaging techniques, to date there are limited data from human studies. Again, one of the challenges for unmodified MSCs is how to identify cells once they have been delivered systemically when there is no idea of location or duration within the recipient tissue; however, when the cells carry a genetic modification then location of small numbers of donor cells within recipient tissues can be more straightforward. There is also a head start when it comes to location of the cells because of the tumor tropism they show.

The TREAT-ME1 trial is attempting to add human data to the literature by delivering modified MSCs to patients after chemotherapy but before surgical resection. Cells will be administered within 72 h of surgery, and samples taken will be analyzed for the presence of the transgene, the activation of the transgene promoter or the transcription of the therapeutic gene. In addition, they will assess for other surrogate markers including tumor neo-angiogenesis, co-localization of modified cells within the tumor site and surrounding tissues and assessment of the tumor microenvironment [104].

In the TACTICAL trial, we are using an image-based approach looking at novel radioisotope labeling that can be detected using positron emission tomography (PET) to enable us to visualize MSCs over multiple time periods. Indium-111 (^111^In) is already routinely used in the clinical setting for white cell and eosinophil labeling and has been assessed as a labeling agent for MSCs. Although MSCs can be labeled successfully with ^111^In, they have low labeling efficiency at approximately 25%, retention within the cells overtime is low and it is more toxic to the cells than it is to leucocytes [111], [112]. In addition, the ^111^In is detected using single-photon emission computed tomography (SPECT) imaging, which does not give as good spatial resolution as tracers that are detected by positron emission tomography scanning. We are currently validating a novel radioisotope and assessing it as a suitable clinical alternative to ^111^In labeling and SPECT imaging. If this agent proceeds to use in the clinical trial, the information gained will further inform future dosing schedules and help to identify any possible off-target effects that would direct potential additional safety monitoring. In addition to the longitudinal imaging, we also propose to collect post mortem samples on patients who give appropriate consent to identify the presence of our allogeneic modified MSCs in tumor samples and to assess both tumor proliferation and apoptosis.

## Summary

Cancer is a devastating disease, and despite some significant advances in cancer therapy, patients who present with advanced, metastatic disease have limited treatment options. Pre-clinical studies using genetically modified stem cells from multiple sources carrying a wide array of therapeutic proteins have shown great promise in the treatment of cancer and have made the progression to first-in-human studies almost inevitable. Even with the ability to build on the great foundations provided by the many clinical trials using unmodified MSCs, the delivery of a genetically modified cell therapy trial remains a significant challenge. The manufacturing process is still one of the greatest barriers to clinical use, and to remain relatively cost-effective, a simple, streamlined process needs to be developed. Although the use of autologous cells undoubtedly has its benefits, the ability to have a cryopreserved allogeneic modified cell therapy will help reduce the inherent stress and costs of manufacturing; to date there is no evidence of immune rejection of this type of product. The knowledge generated from the first two novel clinical trials assessing cell and gene therapy products in patients with cancer will hopefully inform further trials and help to progress this rapidly expanding field and offer great hope for patients with few options.

***Disclosure of interests:*** The authors have no commercial, proprietary, or financial interest in the products or companies described in this article.

## Figures and Tables

**Table I:** Summary of viral vectors used in gene therapy.

Viral vector	Structure	Advantages	Disadvantages
Adenovirus	Double-stranded DNA	DNA incorporated into host cell nucleus Infects dividing and quiescent cells Transient gene expression Lower risk of genotoxicity Large DNA inserts	Transient gene expression Immunogenic Insertional mutagenesis
Adeno-associated virus (AAV)	Single-stranded DNA	Infects dividing and quiescent cells Long-term gene expression Non-cytotoxic Non-immunogenic	Small DNA inserts
Retrovirus	Single-stranded RNA	DNA incorporated into host cell genome Long-term stable gene expression	Insertional mutagenesis Oncogene activation
Lentivirus	Single-stranded RNA	DNA incorporated into host cell genome Long-term stable gene expression Infects dividing and quiescent cells Replication incompetent No insertion into oncogene	

**Table II:** Pre-clinical studies assessing the utility of genetically modified MSCs in cancer.

Tumor type	Therapeutic modification	Cell type	Effects	Ref
Breast	IFN-β	BM-MSC	Reduced tumor growth and metastases and prolonged survival	[73]
Breast	TRAIL	BM-MSC	Reduced tumor growth and metastases	[28], [89]
Lung	PEDF	mBM-MSC	Reduced tumor growth and prolonged survival	[113]
Lung	TRAIL	hUC-MSC	Prolonged survival and increased tumor apoptosis	[114]
Mesothelioma	TRAIL	hBM-MSC	Reduced tumor growth	[26]
Glioma	CDU	hAD-MSC	Tumor regression and prolonged survival	[93]
Glioma	HSV-tK	hAD-MSC	Reduced tumor growth	[94], [115]
Glioma	TRAIL	hUC-MSC	Reduced tumor growth	[22], [116]
Glioma	TRAIL	hBM-MSC	Inhibits tumor growth	[21]
HCC	Apoptin	hBM-MSC	Reduced tumor volume	[92]
HCC	HNF4α	hUC-MSC	Reduced tumor growth	[117]
HCC	IFN-β	hBM-MSC	Decreased tumor formation	[118]
HCC	HSV-tK	mBM-MSC	Reduced tumor growth	[103]
Pancreas	HSV-tK	mBM-MSC	Reduced tumor growth and metastases	[31]
Ascites	IL-12	mBM-MSC	Reduced ascites volume and prolonged survival	[119]
Lymphoma	IL-21	mBM-MSC	Delayed tumor development and prolonged survival	[120]
Prostate	IFN-β	hBM-MSC	Reduced tumor weight and prolonged survival	[121]
